# Evacuation of Pedestrians with Two Motion Modes for Panic System

**DOI:** 10.1371/journal.pone.0153388

**Published:** 2016-04-07

**Authors:** You Zou, Jiarong Xie, Binghong Wang

**Affiliations:** 1 Department of Modern Physics, University of Science and Technology of China, Hefei Anhui, 230026, P. R. China; 2 College of Physics and Electronic Information Engineering, Wenzhou University, Wenzhou, Zhejiang 325035, P. R. China; 3 School of Science, Southwest University of Science and Technology, Mianyang, Sichuan, 621010, P. R. China; Tianjin University, CHINA

## Abstract

In this paper, we have captured an underlying mechanism of emergence of collective panic in pedestrian evacuations by using a modification of the lattice-gas model. We classify the motion of pedestrians into two modes according to their moods. One is gentle (mode I), the other is flustered (mode II). First, to research the cause for crowd, we fix the motion modes of pedestrians and increase the proportion of pedestrians with motion mode II (*ρ*_*II*_). The simulation results show that the pedestrians with motion mode II are lack of evacuation efficiency and cause more casualties. Further, we use the SIS (susceptible-infective-susceptible) model to describe the spreading of the panic mood. The system can be in the high-mix state when the infection probability *λ* is greater than a fuzzy threshold. In addition, the distances *S* from wounded people to the exit are researched, the number of wounded people gets maximum at the internal *S* = 5 ∼ 10, which is independent of *ρ*_*II*_ and *λ*. This research can help us to understand and prevent the emergence of collective panic and reduce wounds in the real evacuation.

## Introduction

The last decades have witnessed a large amount of research on dynamics of pedestrians. It is a complex system containing many bodies and strong relationships among them. Among the various models, three models of them attract special attentions. Hydromechanics [1], social force [2–4], and lattices-gas [5–7] are employed to mimic the dynamical processes of crow flow. In recent years, variant modifications are introduced to these models to make the simulations become closer to the reality [8–16].

The pedestrian evacuation is one of the important aspects of the pedestrian dynamics. In the process of evacuation, the local pedestrian density is much higher and the interactions among pedestrians are stronger and more frequent than the normal pedestrian system. Arching and clogging are observed at exits and the physical interactions in the jammed crowd add up and cause danger. The pedestrian evacuation dynamics were extensively studied in order to determine how to avoid injuries and deaths in emergencies. In 2000, Helbing *et al.* did systematic studies of panic behaviour and quantitative theories capable of predicting the evacuation dynamics based on social force model [17]. In 2001, Yusuke Tajima and Takashi Nagataniand first used the lattice-gas model to simulate the crowd flow going outside a hall and got the relation between evacuation efficiency and size of door [18]. After these milestone works, researchers proposed many modified models to simulate real pedestrian evacuations [19–25].

The present works mainly focus on the interaction of pedestrians, while the collective panic has formed. In this paper, we care about the mechanism of emergence of collective panic in the pedestrian evacuation. We classify the motion of pedestrians into two modes according to their moods. One is gentle (mode I), the other is flustered (mode II). We find that the pedestrian with motion mode II is the initiator of poor evacuation efficiency. Further, the flustered mood could spread from individual to collective. We use the SIS model to describe the spreading of the panic mood and find that there exists a fuzzy threshold of infection probability which renders the system in a high-mix state. The propagation of the panic mood in our model shows an underlying mechanism of emergence of collective panic, which can help us to understand and prevent the emergence of collective panic.

## Model

**Fig 1:**
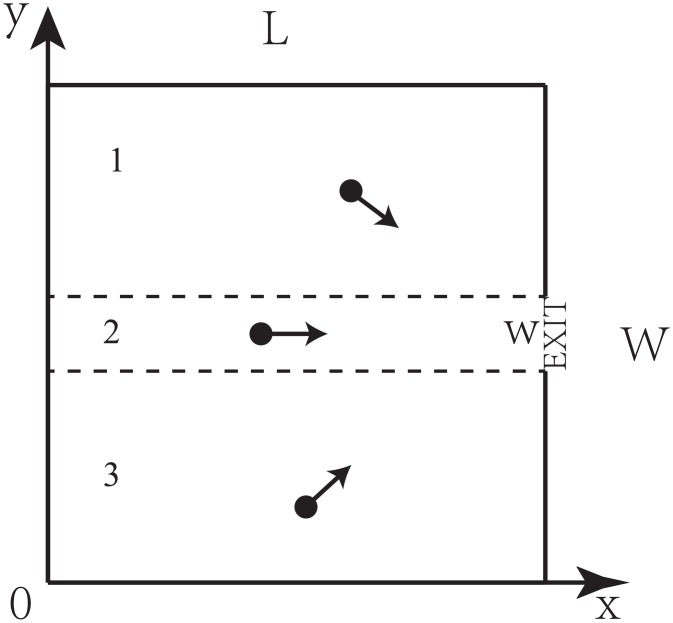
Schematic presentation of the different regions in the system.

The model is studied on a square lattice with size *W* × *L*, where *W*(*L*) is the width (length) of the lattice. To simulate the evacuation process, an exit with width *w* is set at the east border (Fig 1). Each site can be occupied by one pedestrian or be empty. Initially, *N* pedestrians are randomly distributed to the lattice. In each simulation time, each pedestrian can move one step to the site in the potential direction. It is easy for pedestrians to know the approximate location of the exit in real evacuations, so pedestrians do not choose the westward movement in our model. The choice of direction is biased due to the wish of pedestrian to escape, but it is difficult for pedestrians to know their accurate position in real evacuations, so pedestrians could make some bad choices with small probability. If the number of accessible positions is more than one, the pedestrians have higher probability to choose the site closer to the exit. The probabilities of different choices are shown in Table 1. *P*_1_, *P*_2_ and *P*_3_ are the probabilities of the pedestrians choosing the east, north and south site. *D* indicates the strength of the drift. *D* = 1 means pedestrians choose the sites closer to the exit only and *D* = 0 means pedestrians have no preference. If there is only one accessible site, the pedestrian moves into it no matter the choice is good or bad.

**Table 1:** The probabilities of the pedestrians choosing the east(*P*_1_), north(*P*_2_) and south(*P*_3_) site in different regions and cases.

Region	P	Case 1	Case 2	Case 3	Case 4
In the region 1	*P*_1_	$d_{1}+\frac{\left(1-D\right)}{3}$	$D+\frac{\left(1-D\right)}{2}$	$d_{1}+\frac{\left(1-D\right)}{2}$	0
	*P*_2_	$\frac{\left(1-D\right)}{3}$	$\frac{\left(1-D\right)}{2}$	0	$\frac{\left(1-D\right)}{2}$
	*P*_3_	$d_{2}+\frac{\left(1-D\right)}{3}$	0	$d_{2}+\frac{\left(1-D\right)}{2}$	$D+\frac{\left(1-D\right)}{2}$
In the region 2	*P*_1_	$D+\frac{\left(1-D\right)}{3}$	$D+\frac{\left(1-D\right)}{2}$	$D+\frac{\left(1-D\right)}{2}$	0
	*P*_2_	$\frac{\left(1-D\right)}{3}$	$\frac{\left(1-D\right)}{2}$	0	1/2
	*P*_3_	$\frac{\left(1-D\right)}{3}$	0	$\frac{\left(1-D\right)}{2}$	1/2
In the region 3	*P*_1_	$d_{1}+\frac{\left(1-D\right)}{3}$	$d_{1}+\frac{\left(1-D\right)}{2}$	$D+\frac{\left(1-D\right)}{2}$	0
	*P*_2_	$d_{2}+\frac{\left(1-D\right)}{3}$	$d_{2}+\frac{\left(1-D\right)}{2}$	0	$D+\frac{\left(1-D\right)}{2}$
	*P*_3_	$\frac{\left(1-D\right)}{3}$	0	$\frac{\left(1-D\right)}{2}$	$\frac{\left(1-D\right)}{2}$

That the three neighbor sites are accessible positions is labeled as Case 1. Case 2, Case 3 and Case 4 display the cases of the south, north and east neighbor site is unaccessible respectively. Here, $d_{1}=D\times \frac{L-x_{i}}{|y_{i}-W/2|+\left(L-x_{i}\right)}$, $d_{2}=D\times \frac{|y_{i}-W/2|}{|y_{i}-W/2|+\left(L-x_{i}\right)}$. (*x*_*i*_, *y*_*i*_) is the coordinate of the pedestrian *i*.

Herein we try to keep the model as simple as possible. This effort may lead to few unreasonable choices (bugs). For instance, when a pedestrian is very close to or even reaches the east boundary, the better choice becomes obvious but he/she will still choose the bad position with a certain probability.

There are two different motion modes of the pedestrians in our model. The pedestrians with mode I can only move into an empty site. The empty neighbor sites except the westward site are their accessible positions. They can not move when the three potential neighbor sites are all occupied. The pedestrians with mode II are flustered. They can move to not only an empty site, but also an occupied site. After choosing a neighbor site, the pedestrian *i* wants to move into it. If the chosen site is occupied, *i* can also move into it and forces the one in the site to exchange position with *i* with probability *p*. If the motion mode of the pedestrian at the chosen site is mode II, *p* = 0.5, otherwise *p* = 1.

The procedure is done with parallel update rule. When two or more pedestrians choose the same site, the site will be occupied by one of them randomly. After pedestrian escapes, he/she will be removed from the system. In this model, the crowd flow going outside the room is reduced to its simplest form.

## Simulations and results

Simulations are carried out on a square lattice for a room scale 25 × 25, the width of the exit is *w* = 3 and the pedestrian scale is *N* = 500. We set the strength of the drift *D* = 0.6.

### Situation I (motion mode fixed)

**Fig 2:**
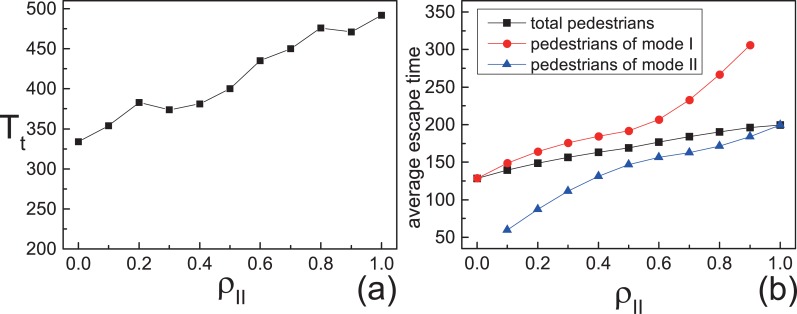
The escape time of fixed motion mode. All the data points above are obtained by averaging over 500 different realizations. (a)The escape time *T*_*t*_ that all the pedestrians escape from the room. (b)Average escape time as functions of *ρ*_*II*_.

**Fig 3:**
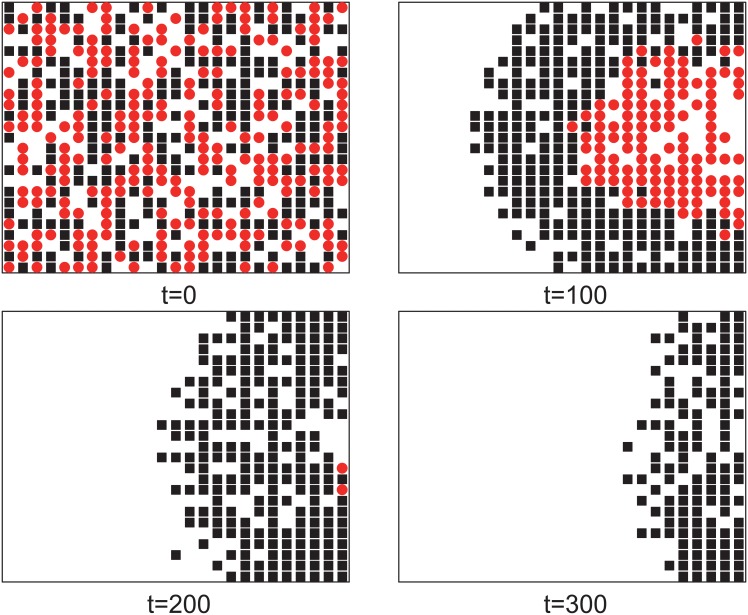
Typical pattern of evolution of pedestrians on square lattice 25 × 25. The pedestrians with motion mode I are indicated by black squares, and the pedestrians with motion mode II are indicated by red circles. Here, *ρ*_*II*_ = 0.5.

In this situation, the motion modes of pedestrians are fixed by initial random assignment. The initial proportion of pedestrians with motion mode II is labeled *ρ*_*II*_. From Fig 2(a), we can see that the escape time *T*_*t*_ that all the pedestrians escape from the room increases with *ρ*_*II*_. From another perspective, the average escape time $\bar{T}$ ($\bar{T}=\frac{1}{N}\sum t_{i}$, *t*_*i*_ is the time that the pedestrian *i* escaped from the room) increases with *ρ*_*II*_ too. These results show that most individuals evacuated slowly when *ρ*_*II*_ is large. But the average escape time of pedestrians with motion mode II is shorter than that of pedestrians with motion mode I (shown in Fig 2(b)). A typical pattern of evolution of pedestrians is shown in Fig 3. In the beginning, the pedestrians with different motion modes are randomly assigned to the lattice. Later, the pedestrians with motion mode II form an arch-shaped cluster around the exit and separate from the pedestrians with motion mode I. After most pedestrians with motion mode II escaped, the pedestrians with motion mode I could exit from the room. The above shows that the individual aggressive behavior is beneficial to the pedestrians with it but is harmful to the overall efficiency.

The pedestrians with motion mode II may exchange position with their neighbor. The exchanges give rise to wounds. If a pedestrian *i* with motion mode II exchanges the location with *j*, *j* would be wounded with small probability. We assume that *p*_*w*,*I*_ (the probability of pedestrians with motion mode I becoming wounded) is greater than *p*_*w*,*II*_. A wounded person does not move or exchange position, and becomes a barrier. Because of the existence of the barriers, some healthy pedestrians are obstructed and some pedestrians even get stuck. In our simulations, if people can not escape before *t* = 5000, we regard them as stranded people.

**Fig 4:**
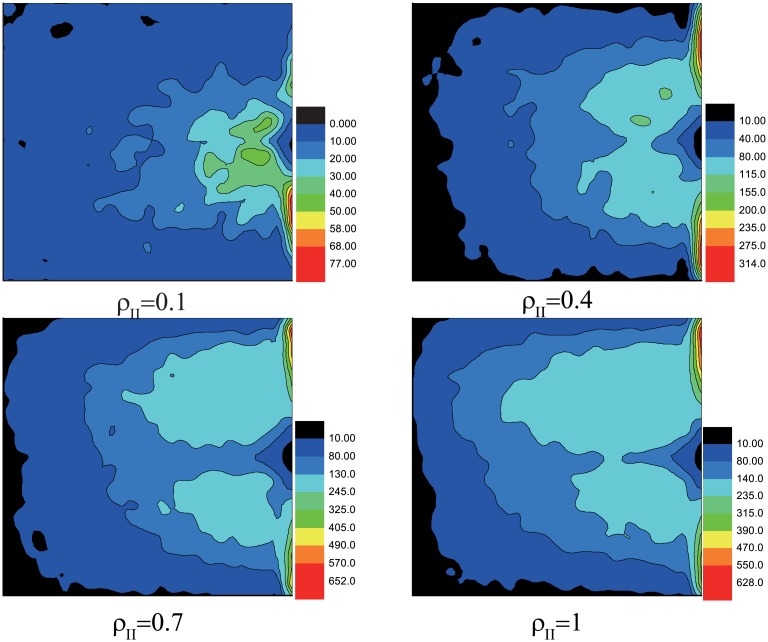
The spatial distribution of the wounded people with different *ρ*_*II*_. The color reflect the number of accumulated wounded people in different area. Here, the scale of the room is 25 × 25, *w* = 3 and *N* = 500. The data above are obtained by 5000 different realizations.

**Fig 5:**
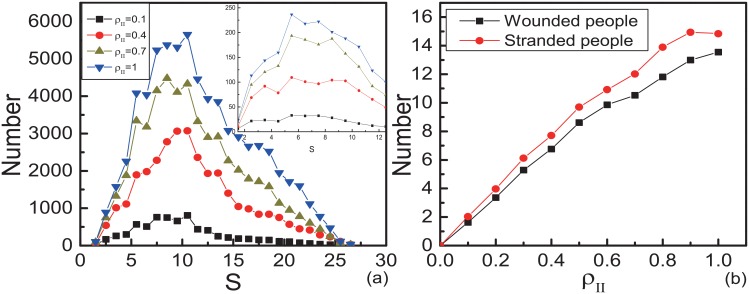
Number of wounded and stranded people with fixed motion mode. Here, *p*_*w*,*I*_ = 0.001 and *p*_*w*,*II*_ = 0.0001. (a)The distribution of the distances from wounded people to the exit. The inset graph displays the distribution of the number of wounded people per unit area. The horizontal ordinate of every points *S* is the median of internal [*S* − 0.5, *S* + 0.5]. The data are obtained by 5000 different realizations. (b)The numbers of wounded people and stranded people as functions of *ρ*_*II*_. The data points are obtained by averaging over 500 different realizations.

The spatial distribution of the wounded people with different *ρ*_*II*_ is shown in Fig 4. There exists three high-incidence areas of wounds, one is in the middle, the others are on the two sides of the exit. The wounded people will block the pedestrians at the east edge. Those stranded people are more likely to be wounded by long time retention, so the two high-incidence areas of wounds at the east edge form. The distance *S*_*i*_ from wounded pedestrian *i* to the exit is researched. We count the number of wounded in the internal [*S* − 0.5, *S* + 0.5]. The distribution of the wounded people is shown in Fig 5(a). The inset graph in Fig 5(a) displays there exists a peak at the same internal *S* = 5 ∼ 10 with different *ρ*_*II*_. From the simulated results, the areas most likely to give rise to wounds can be founded. In the reality, we should pay more attention (such as arranging control staffs) to those areas to reduce the number of wounded.

Fig 5(b) shows that the number of the wounded increases as *ρ*_*II*_ increases. Even *p*_*w*,*I*_ and *p*_*w*,*II*_ are very small, a mass of exchange actions cause non-ignorable number of accidents. All the above results show that the pedestrians with motion mode II make the evacuation efficiency lower and cause more casualties.

### Situation II (motion mode variable)

In real evacuations, the emotions of pedestrians are not fixed. The gentle pedestrians would become flustered due to the influence of the emotions and actions of their neighbors. In addition, when the pedestrians are exhausted or think that the danger is gone, they could be gentle again. The process is much like the classical rumor propagation process [26–30]. Here, we use the SIS (susceptible-infective-susceptible) model [29, 30] to simulate the changes between mode I and mode II. The probability that a pedestrian *i* with motion mode I changes to mode II under the influence of each neighbor with motion mode II is *λ*. If the number of his neighbors with motion mode II is *n*, the probability that motion mode of *i* changes to mode II at next step is *P* = 1 − (1 − *λ*)^*n*^. In each time step, the probability that pedestrians change their motion mode from II to I is *β*. In this paper, we fix *β* = 0.1.

**Fig 6:**
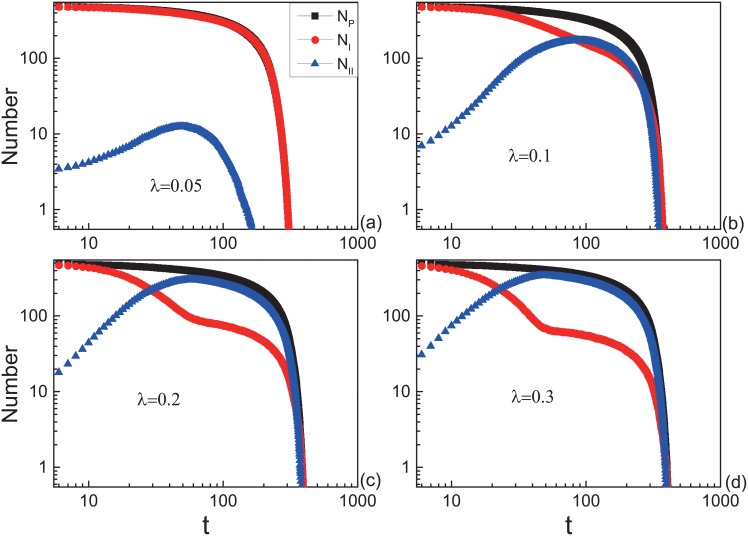
Evolution of the number of pedestrians (*N*_*p*_), the number of pedestrians with motion mode I (*N*_*I*_) and mode II (*N*_*II*_) in the system for different *λ*. All the data points above are obtained by averaging over 500 different realizations. Initially, *N*_*II*_ = 5.

First, we consider the evacuation without wounded. When *λ* is small, the number of pedestrians with motion mode II (*N*_*II*_) reduces to zero fast, it means that the panic could not spread widely. Increasing the *λ*, the peak of the *N*_*II*_ gets close to the *N*_*p*_. Large *λ* gives rise to high proportion of pedestrians with mode II. The result is shown in Fig 6. There is a remarkable difference between the situations of *λ* ≥ 0.2 and *λ* < 0.2. When *λ* ≥ 0.2, the peak of *N*_*II*_ is close to *N*_*p*_ and *N*_*II*_ reaches the peak before the rapid decrease of *N*_*p*_ (see Fig 6(c) and 6(d)). While *λ* < 0.2, the peak of *N*_*II*_ is not close to *N*_*p*_ (see Fig 6(a)) or *N*_*II*_ closes to *N*_*p*_ after the rapid decrease of *N*_*p*_ (see Fig 6(b)).

**Fig 7:**
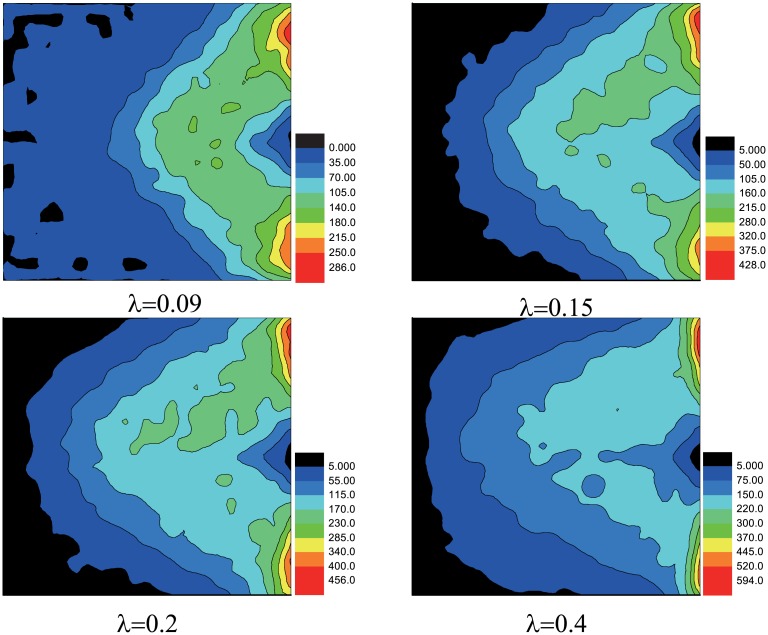
The spatial distribution of the wounded people with different *λ*. The color reflect the number of accumulated wounded people in different area. Here, the scale of the room is 25 × 25, *w* = 3 and *N* = 500. Initially, *N*_*II*_ = 5. The data above are obtained by 5000 different realizations.

**Fig 8:**
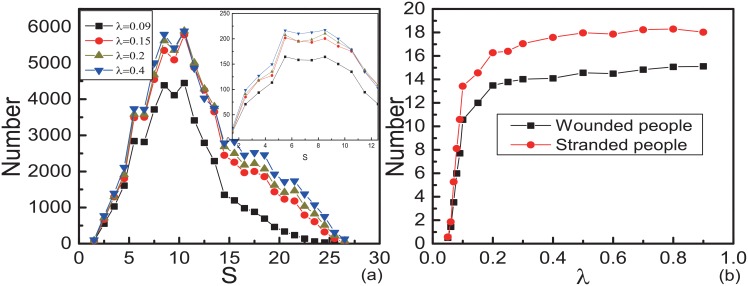
Number of wounded and stranded people with variable motion mode. Here, *p*_*w*,*I*_ = 0.001 and *p*_*w*,*II*_ = 0.0001. Initially, *N*_*II*_ = 5. (a)The distribution of the distances from wounded people to the exit. The inset graph displays the distribution of the number of wounded people per unit area. The data are obtained by 5000 different realizations. The horizontal ordinate of every points *S* is the median of internal [*S* − 0.5, *S* + 0.5]. (b)The numbers of wounded people and stranded people as functions of *λ*. The data points are obtained by averaging over 500 different realizations.

To find its essential, we study the propagation model with wounded. The spatial distribution of the wounded people with different *λ* is shown in Fig 7. Fig 8(a) displays the number of them takes maximum value at the same interval *S* = 5 ∼ 10 with different *λ*. Comparing with Figs 4 and 5(a), we find the statistical results are much similar no matter the motion mode fixed or not. In social force model, pedestrians near the exit get strongest pressure and have the maximum probability to be wounded [17]. In real situations, pedestrians near the exit may be extruded hard, but they can get out of the room in a short time, so the possibility of them becoming wounded gets smaller instead. Our results can explain this phenomenon.

**Fig 9:**
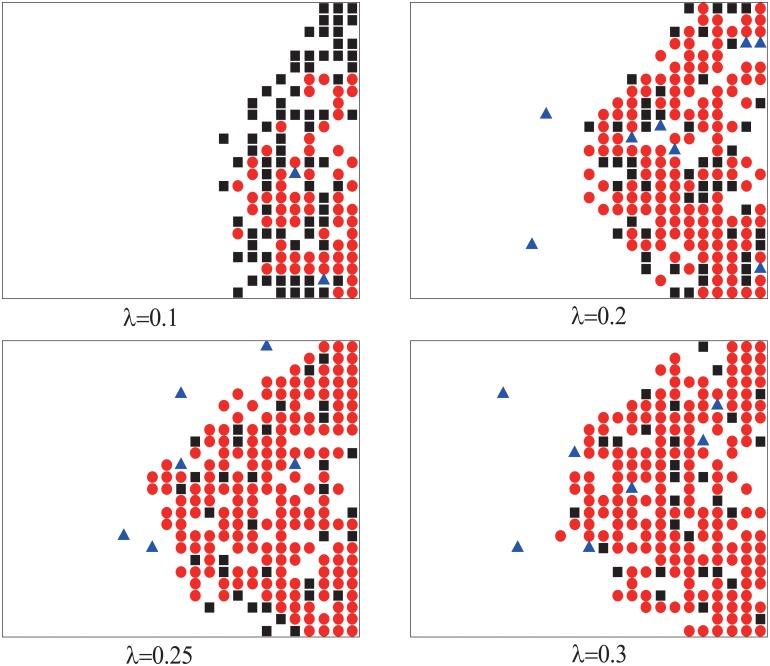
Typical pattern of distribution of pedestrians on the square lattice 25 × 25 for different *λ* when *t* = 300. The pedestrians with motion mode I are indicated by black squares, the pedestrians with motion mode II are indicated by red circles, and the wounded people are indicated by blue triangles. Initially, *N*_*II*_ = 5.

From Fig 8(b), we can see that the numbers of wounded people and stranded people are non-linear relation with *λ*. When *λ* = 0.2, the number of wounded people is almost saturated. Comparing with Fig 5(b), we can see that the saturated number of wounded people here is larger than that when motion modes of all the pedestrians are mode II fixed. There are two reasons. One is when pedestrian *i* with mode II attempts to exchange position with pedestrian *j*, the probability of position exchange depends on the motion mode of *j*. The mixture of pedestrians with two motion modes leads to more exchanges. The other is *p*_*w*,*I*_ > *p*_*w*,*II*_, more wounded will appear with exchanges between pedestrians with different modes than same. In Fig 9, when *t* = 300, the different distributions of pedestrians on the square lattice are shown. The pedestrians of different motion modes do not separate. When *λ* is large enough, the pedestrians with motion mode II become dominant and most pedestrians with motion mode I are surrounded by the pedestrians with motion mode II. These explain why there are more wounded people with motion mode variable.

**Fig 10:**
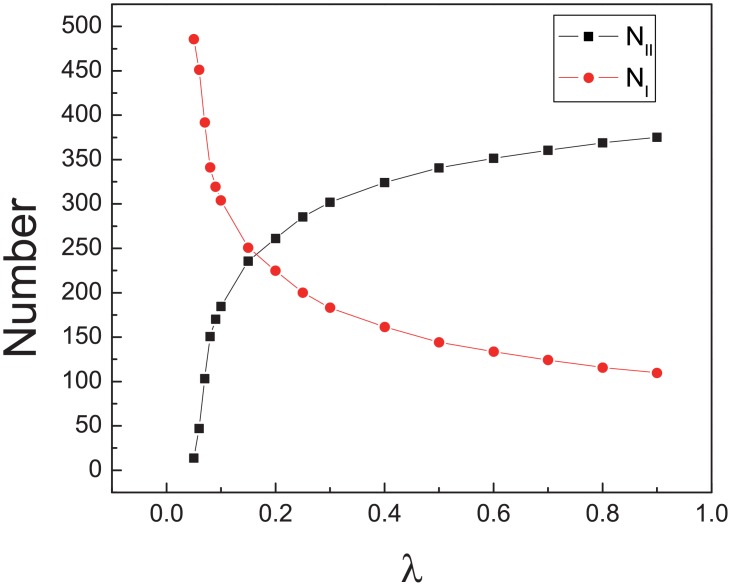
The statistics of the escaped pedestrians. *N*_*I*_ and *N*_*II*_ are the number of pedestrians with motion mode I and mode II when pedestrians get out of the room. All the data points above are obtained by averaging over 500 different realizations. Initially, *N*_*II*_ = 5.

On the other hand, the statistics of the motion mode of pedestrians when they get out of the room can help us to understand the results above (see Fig 10). *N*_*I*_ reduces and *N*_*II*_ increases when *λ* increases. But when *λ* ≥ 0.2, the gradients of *N*_*I*_ and *N*_*II*_ are much smaller.

The pedestrian evacuation system possesses some attractive features when *λ* ≥ 0.2. The proportion of motion mode II pedestrians is high and they dominate the system. The two kinds of pedestrians mix heavily and form an arch-shaped cluster around the exit. The numbers of wounded and stranded are almost saturate. We think the high level of mix is the essential of this state. We define it as the high-mix state and *λ*_*c*_ = 0.2 is the fuzzy threshold. Small *λ* can suppress the formation of the high-mix state. In this state, the system is chaotic and causes many wounded. We hope the system is not in this state. The large *λ* is harmful to the evacuation. In reality, reducing *λ* would prevent the emergence of collective panic.

**Fig 11:**
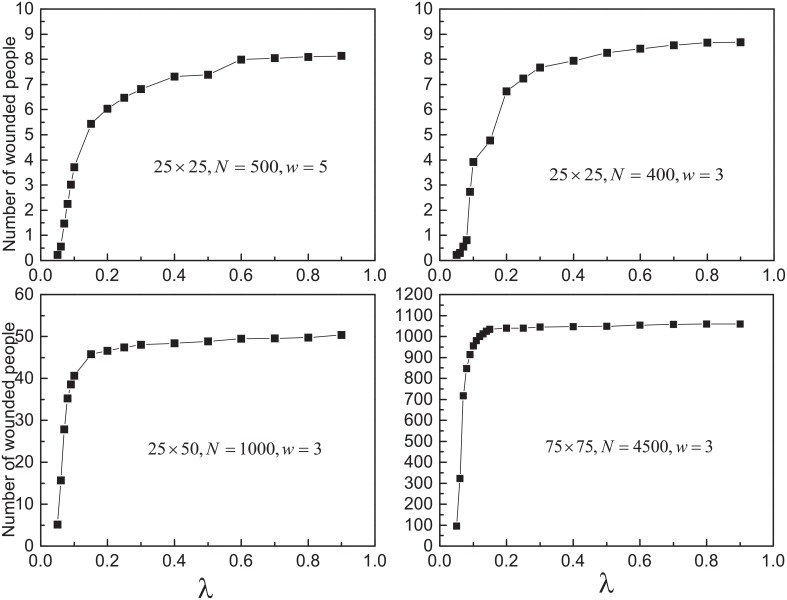
The number of wounded people as function of *λ* with various parameters. All the data points above are obtained by averaging over 500 different realizations. Initially, *N*_*II*_ = 5.

Further more, the impact of parameter values on the results has been studied. We change the size of the exit, pedestrian density, the shape and size of the room. The relationship between the number of wounded people and *λ* with various parameters is shown in Fig 11. There exists a fuzzy threshold in each circumstance. When evacuation conditions become worse, the threshold decreases and the proportion of the saturate wounded people increases.

## Conclusion

In this work, the evacuation of panic pedestrians has been studied based on the modified lattice-gas model. There are two motion modes of the pedestrians. The pedestrians with motion mode I only move into an empty site. The pedestrians with motion mode II are more flustered, and they can force their neighbors to exchange the position with them. We studied the impacts of the initial proportion of motion mode II (*ρ*_*II*_) on the evacuation of the system. The time *T*_*t*_ that all the pedestrians escape from the room and the average escape time $\bar{T}$ both increase with increasing *ρ*_*II*_. The number of wounded people increases as *ρ*_*II*_ increases and their function is almost linear. The pedestrian with motion mode II is the initiator of poor evacuation efficiency.

Then, to reflect the mental interaction, we regarded the contagion of emotion as the rumor propagation process. Pedestrians with motion mode I were regarded as susceptible individuals and pedestrians with mode II were regarded as infected individuals. We used the SIS model to simulate the spreading of the panic mood. Large *λ* gives rise to high proportion of pedestrians with mode II. We find the fuzzy threshold *λ*_*c*_ of the system. When the infection probability is above *λ*_*c*_, the system is in high-mix state. The process of individual panic becoming collective panic is shown in our simulation. Our model proposes an underlying mechanism of the emergence of collective panic, it will help us to understand and prevent the emergence of collective panic.

From the spatial distribution of the wounded, we find the number of them takes maximum value at the same interval *S* = 5 ∼ 10, which is independent of *ρ*_*II*_ and *λ*. This find can help us to reduce wounds in real evacuations.
